# A novel intervention for treating adults with ADHD using peripheral visual stimulation

**DOI:** 10.3389/fpsyt.2023.1280440

**Published:** 2023-10-20

**Authors:** Yael Richter, Carlos Gordon, Gabriel Vainstein, Carmel Bublil-Mor, Dario Geisinger, Noya Meital-Kfir, Zohar Elyoseph

**Affiliations:** ^1^VIZO-Specs Ltd., Tel Aviv, Israel; ^2^Kahan-Sagol-Maccabi Research and Innovation Institute, Tel Aviv, Israel; ^3^The Department of Educational Psychology and Educational Counseling, Max Stern Yezreel Valley College, Emek Yezreel, Israel

**Keywords:** ADHD, ADHD intervention, Adult ADHD, peripheral vision, peripheral visual stimulation

## Abstract

**Objective:**

Stimulation of the peripheral visual field has been previously reported as beneficial for cognitive performance in ADHD. This study assesses the safety and efficacy of a novel intervention involving peripheral visual stimuli in managing attention deficit hyperactivity disorder (ADHD).

**Methods:**

One hundred and eight adults, 18–40 years old, with ADHD, were enrolled in a two-month open-label study. The intervention (i.e., Neuro-glasses) consisted of standard eyeglasses with personalized peripheral visual stimuli embedded on the lenses. Participants were assessed at baseline and at the end of the study with self-report measures of ADHD symptoms (the Adult ADHD Self-Report Scale; ASRS), and executive functions (The Behavior Rating Inventory of Executive Function Adult Version; BRIEF-A). A computerized test of continuous performance (The Conners’ Continuous Performance Test-3; CPT-3) was tested at baseline with standard eyeglasses and at the end of study using Neuro-glasses. The Clinical Global Impression-Improvement scale (CGI-I) was assessed at the intervention endpoint. Safety was monitored by documentation of adverse events.

**Results:**

The efficacy analysis included 97 participants. Significant improvements were demonstrated in self-reported measures of inattentive symptoms (ASRS inattentive index; *p* = 0.037) and metacognitive functions concerning self-management and performance monitoring (BRIEF-A; *p* = 0.029). A continuous-performance test (CPT-3) indicated significant improvement in detectability (d’; *p* = 0.027) and reduced commission errors (*p* = 0.004), suggesting that the Neuro-glasses have positive effects on response inhibition. Sixty-two percent of the participants met the response criteria assessed by a clinician (CGI-I). No major adverse events were reported.

**Conclusion:**

Neuro-glasses may offer a safe and effective approach to managing adult ADHD. Results encourage future controlled efficacy studies to confirm current findings in adults and possibly children with ADHD.

**Clinical trial registration**: https://www.clinicaltrials.gov/, Identifier NCT05777785.

## Introduction

Attention deficit hyperactivity disorder (ADHD) poses a significant public health problem estimated to affect up to 11% of children worldwide (1). ADHD is commonly first diagnosed during the early school years due to its substantial role in the emergence of school-related disruptions. Nevertheless, it is a life-long disorder, affecting as many as 7% of the adult population (2). Symptoms of ADHD include inattentive and hyperactive–impulsive behaviors that persistently impact educational, occupational, interpersonal, and emotional functioning (3, 4).

Although ADHD has a wide range of adverse outcomes, it is one of the most treatable neurobehavioral disorders. Stimulants, such as amphetamines and methylphenidates, are a common ADHD treatment with documented safety and efficacy (5, 6). Nevertheless, stimulant treatment is not without limitations. It is frequently associated with bothersome side effects, ambivalence regarding chronic medication usage, and inconvenience or ineffective dosing. More than half of the patients being offered a pharmacotherapy treatment do not follow their medication regimen, even shortly after initiating treatment (7, 8). This low adherence rate and premature treatment termination undermine the benefits of stimulant treatment and support the need for developing new interventions for ADHD.

In this study, we use novel glasses, i.e., Neuro-glasses, as an intervention for treating adults with ADHD. It is worth noting that ADHD has been found to be associated with various visual problems and oculomotor abnormalities (9–12). A recent meta-analysis showed a link between ADHD and atypical corneal curvature (e.g., astigmatism), problems controlling eye muscles (e.g., hyperopia and hypermetropia, and strabismus), reduced contrast sensitivity, and color discriminability (10). Interestingly, ADHD was not directly related to problems in visual acuity, refractive error, or anatomic ocular measures. These findings underscore the necessity for including visual assessments within the scope of ADHD evaluations. Impaired visual perception has a direct impact on attention and cognition. Consequently, if left untreated, vision deficits could potentially worsen existing ADHD symptoms or even manifest as ADHD-like symptoms, leading to a false diagnosis of ADHD and vice versa. It is essential to emphasize, however, that the Neuro-glasses used in this study are not an optometry solution by its very nature. Rather, their impact on attention-related performance is induced using stimuli in the peripheral visual field.

Peripheral vision provides essential information concerning our surroundings. It has a survival function as it guides and relates us to the world around us, showing high sensitivity to movement and brightness changes in the visual scene (13, 14). Although the vast majority of the human retina is devoted to peripheral vision, it is frequently considered uninformative compared to foveal, detailed-oriented vision. Indeed, peripheral vision provides low acuity and poor color vision, however, much of our perception relies on peripheral inputs (13–16). Among other systems, peripheral vision is involved in balance, movement, and stress. A wide range of everyday tasks, such as driving, sports, visual searching, or even walking, do not demand high acuity vision but mainly depend on our ability to see outside the point of fixation (14–16).

Given the key role of peripheral vision in monitoring the environment and facilitating rapid detection of changes in the visual scene, it is expected to affect the allocation of attentional resources. Peripheral vision allows us to react quickly and shift attention to adjust our visually guided behavior to the visual scene. Without sufficient peripheral vision providing the visual context in which objects exist, it is difficult to distinguish relevant from nonrelevant information (14, 17–19). Peripheral vision provides contextual input that helps prioritize the regions in the visual scene relevant to a given task. Based on which the attentional resources are allocated (13–15).

Previous studies have shown that peripheral stimuli external to a task often influence performance (17, 19–21). Facilitation was reported to depend on the features of the stimulus, its relevance to the task, and its novelty (20). Increased sensitivity to peripheral stimuli was reported for nonclinical adults with ADHD-like traits (22). Participants with high levels of ADHD-like traits were more responsive than control to the cueing role of peripheral stimuli, indicating the arrival of a target. This elevated sensitivity allowed the participants with high levels of ADHD-like traits to allocate attentional resources more efficiently to meet task demands. The assumption was that facilitation in ADHD reflects hypersensitivity of the superior colliculus (SC), a structure concerned with peripheral vision (22, 23). However, accumulating evidence indicates that ADHD patients benefit from extra-task stimuli even when they are centrally presented, involve other modalities (i.e., auditory, motor information), or lack informational content (22, 24–27). For example, white noise exposure was highly beneficial for individuals with ADHD (24, 28, 29). A possible explanation is that stimuli external to a task operate via an additive effect, where the addition of noise boosts a naturally too-weak signal above a threshold level, allowing its detectability (28, 29).

The beneficial effect of glasses using peripheral visual stimulation was previously reported in patients with chronic dizziness (30). It was assumed that constant visual cues on the peripheral visual field strengthen the information of real head motion, possibly reducing the mismatch between sensory and locomotor information. Here, we hypothesize that the cueing effect of peripheral vision stimulation is not unisensory and affects a broad spectrum of attention-based performances. This study aims to assess the efficacy and safety of peripheral stimulation glasses, the Neuro-glasses, in adults with ADHD. It is further hypothesized that by stimulating the visual periphery, the Neuro-glasses will attenuate ADHD symptoms and related functions.

## Materials and methods

### Participants

Eligible participants included adults aged 18 to 40 with a documented history of ADHD diagnosis. The upper age limit was predetermined to avoid age-related cognitive and visual decline. Exclusion criteria were: 1) any psychiatric or neurological comorbidity (e.g., epilepsy, Autism, depression, traumatic brain injury), 2) concomitant use of ADHD medications (i.e., stimulants or non-stimulants) 4 weeks before entering the study, and 3) undergoing neurofeedback or cognitive training treatment.

### Trial design

This was a two-month, open-label study in which eligible ADHD-diagnosed adults were provided with a pair of Neuro-glasses featuring a personalized visual stimuli pattern for each participant. A baseline assessment of the participants’ ADHD clinical profile pre-intervention included the Adult ADHD Self-Report Scale (ASRS; 31) and the Behavior Rating Inventory of Executive Function Adult Version (BRIEF-A; 32) questionnaires. In addition, participants completed the Conners’ Continuous Performance Test-3 (CPT-3; 33) while wearing standard eyeglasses without stimuli at the peripheral visual field. Optical centration parameters and demographic information were collected for each participant. Participants were then invited to complete a personalization process in which they were fitted with personalized Neuro-glasses (see ‘Neuro-glasses intervention’ section for detailed description). Participants were instructed to wear their Neuro-glasses for at least 2 hours daily for 2 months.

**Figure 1:**
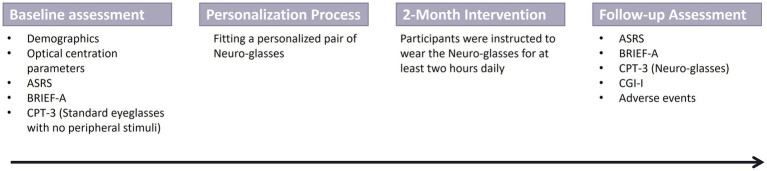
Schematic illustration of the study design.

A follow-up assessment at the end of the intervention re-assessed ADHD performance on the ASRS and BRIEF-A questionaries. In addition, participants completed the CPT-3 test while wearing their Neuro-glasses.The Clinical Global Impression-Improvement (CGI-I; 34) rating scale was administered by a clinician (Figure 1).

### Neuro-glasses intervention

**Figure 2:**
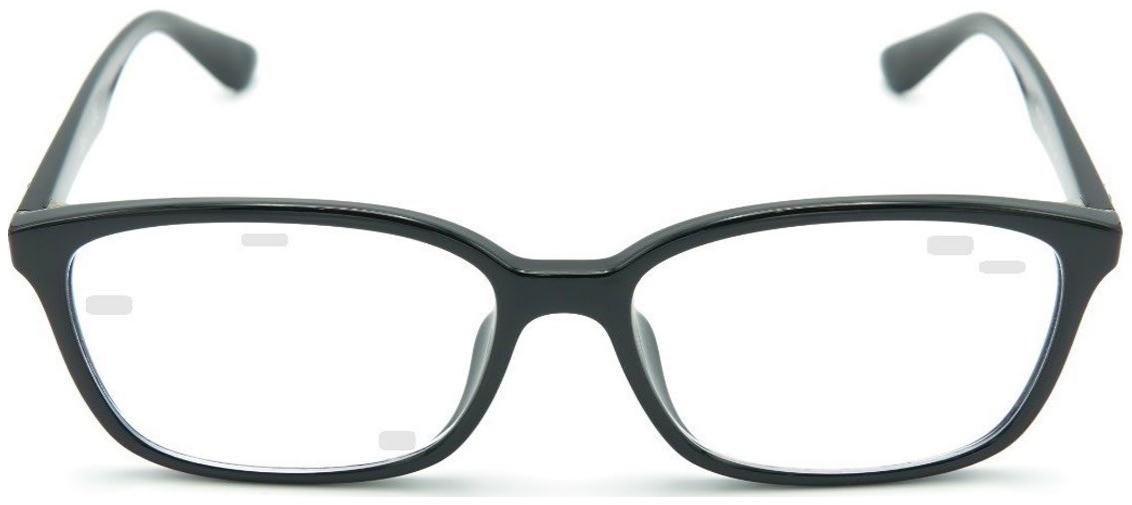
Illustration of a sample Neuro-glasses consisting of a standard eyeglasses frame and standard optic lenses with semi-transparent marking stimuli embedded on the lenses.

The Neuro-glasses (Sparkles^™^, supplied by VIZO Specs Ltd.) consisted of a standard eyeglasses frame and standard optic lenses with semi-transparent marking stimuli, a few millimeters in size, embedded on the lenses (Figure 2). In cases where prescription glasses were needed, the Neuro-glasses were corrected to copy the participant’s existing prescription.

The stimuli pattern was adjusted at VIZO’s facility to fit the optimal performance of an individual. This personalization process included three visits. In each visit, participants completed a proprietary battery of neurocognitive tests while wearing eyeglasses with different stimuli patterns. The tests examined performance on tasks of selective attention, endogenous and exogenous attention, shifting of attention, processing speed, visual search, and inhibition of response. The participants were tested in front of an eye tracker (Tobii Pro Spectrum, Tobii, Inc., Sweden) sampling at 150 Hz with a 23.8-inch display mounted on top (EIZO FlexScan EV2451, EIZO Inc., United States) and viewed at 60 cm distance. The tests were carried out using E-Prime 3.0 (Psychology Software Tools Inc., United States) running on a standard personal computer (Intel i7, 64Gb RAM, NVIDIA 1080Ti running Windows 10). Eye-tracking parameters such as oculomotor reaction time and pupil size, as well as behavioral measures of time and accuracy, were collected and processed by VIZO’s proprietary cloud-based system to determine the optimal number and size of the stimuli and their positioning in the peripheral visual field.

### Outcome measures

#### Efficacy measures

The outcome measures provide a broad assessment of ADHD symptoms, executive functions, global functioning, and measures of attention and impulsivity. The assessment involved self-report questionnaires, clinician evaluation, and a task-oriented computerized assessment.

The Adult ADHD Self-Report Scale (ASRS; 31) is a self-report assessment comprising 18 items corresponding to the DSM-V-TR criteria for ADHD. The ASRS is a self-administered instrument whereby participants rate 18 symptom items using a 5-point Likert scale ranging from 0 (‘Never’) to 4 (‘Very Often’). The total score ranges between 0 to 72, with higher scores indicating greater ADHD impairments. The ASRS total score is comprised of two subscales: Inattention and Hyperactivity.

The Behavior Rating Inventory of Executive Function Adult Version (BRIEF-A; 32) is a standardized measure that captures views of adults’ executive functions or self-regulation in their everyday environment. The BRIEF-A comprises 75 items, each rated by the individual, using a 3-point Likert scale ranging from 0 (‘Never’) to 2 (‘Often’). The total score ranges between 0 to 150. BRIEF-A includes a Behavioral Regulation index, a Metacognition index, and a summary score-Global Executive Composite.

The Clinical Global Impression-Improvement (CGI-I; 34) provides an overall clinician-rated summary measure of improvement on a 7-point scale, ranging from 1 (very much improved) to 7 (very much worsened).

The Conners’ Continuous Performance Test-3 (CPT-3; 33) is a computerized task of attention and impulsivity. It includes a serial presentation of target and non-target letters. Participants are instructed to press a button when any letter, except the letter “X” (i.e., target), is presented on the screen. The CPT-3 provides numerous summary measures: 1) d-prime (d’) – measures “signal detectability,” that is, the respondent’s ability to differentiate non-targets (i.e., the letter X) from targets (i.e., all other letters); 2) Omissions – missed targets; 3) Commissions – incorrect responses to non-targets (False alarm); 4) Reaction Time (HRT) – the mean response speed; 5) Hit Reaction Time Standard Deviation (HRT SD) – the consistency of response speed to targets.

#### Safety and feasibility measures

To assess adherence, participants were instructed to document their daily usage of the eyeglasses (i.e., total hours per day). Potential side effects and adverse events were assessed by the study investigators during the follow-up assessment.

### Statistical analyzes

Paired samples *t*-tests were used to test the effect of the Neuro-glasses intervention, comparing the baseline and end-of-intervention performances. Normality distribution of data was assessed using the Shapiro–Wilk test. Due to the exploratory nature of the study, no corrections for multiple testing were applied. The safety analysis included all available data on the intent-to-treat (ITT) population.

Mixed model ANOVA was used to explore any potential effect of covariates such as gender, age, eyeglasses, learning disabilities, treatment naïve, and ASRS total score. None of the covariates were found significant, thus were not included in the final model.

Power analyzes determined that a sample size of 90 participants will have 80% power to detect an effect size of 0.30 (Cohen’s *D*) using a paired t-test with a 0.05 two-sided significance level. Assuming a 20% attrition rate over the course of the study, 108 participants were targeted for inclusion (35). This relatively small effect size was determined given the heterogeneity of the study population, as participation was neither limited by the severity nor occurrence of ADHD symptoms. All analyzes were conducted using SPSS^®^ Version 26.

## Results

### Study population

**Figure 3:**
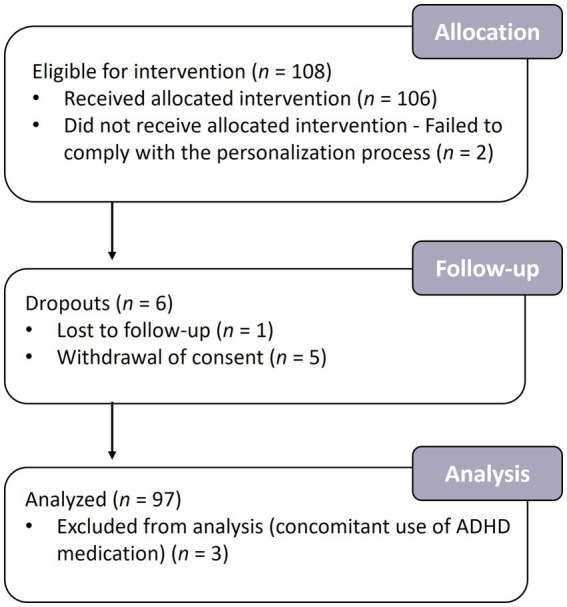
Flowchart of the participants in the study. A total of 108 participants were enrolled in the study, of whom 100 completed the intervention and 97 were analyzed.

Of 119 individuals screened, 108 met the inclusion criteria. Two participants failed to comply with the personalization process due to difficulty staying awake or using a cell phone during the test, resulting in 106 participants receiving the intervention. Five participants voluntarily withdrew consent at the end of the intervention, and one was lost to follow-up (Figure 3). One hundred participants completed the study; three were excluded from the efficacy analysis due to a major protocol deviation (concomitant use of ADHD medication). The per-protocol (PP) population included the remaining 97 participants. Participants’ demographics are summarized in Table 1.

**Table 1:** Demographics and baseline characteristics of the per-protocol cohort (*n* = 97).

Demographics / Baseline characteristics	
Age [Mean (SD)]	26.51 (4.59)
ASRS Inattentive subscale [Mean (SD)]	21.21 (6.64)
ASRS Hyperactive subscale [Mean (SD)]	18.60 (6.58)
ASRS Total baseline [Mean (SD)]	39.81 (12.00)
Gender (% male)	47%
Treatment-Naive (% naive)	10%
Learning disability history (%)	64%
Eyeglasses (%)	23%

### Safety and tolerability

The Neuro-glasses were well tolerated. No serious adverse events were reported throughout the two-month intervention, and no events led to intervention discontinuation. All side effects were transient and reported to be mild or moderate. Of the 100 participants, only 65 reported one or more adverse events. The remaining 35 participants had no adverse events (Table 2). The occurrence of adverse events was not significantly different between participants having prescription eyeglasses than those without a history of prescription eyeglasses.

**Table 2:** Percentage of participants reporting intervention-related adverse events (*n* = 100).

	% Reporting
Total intervention-related adverse events^a^	65
Headache	36
Eyestrain	20
Dizziness	18
Tiredness	9
Discomfort	7
Visual discomfort	3
Nausea	3
Feeling hyper	1

a The “total intervention-related adverse events” reflects the percentage of participants experiencing any adverse event judged as related or probably related to the intervention. Thus, participants who experienced more than one adverse event were counted only once.

Adherence results indicate high acceptability. Sixty-three percent of the participants reported wearing the glasses for at least 4 hours daily, whereas only 7% wore the glasses for less than 2 hours daily (Table 3).

**Table 3:** Usage time per day (*n* = 97).

Usage time (hours)	Reporting (%)
> 6	34
4–6	29
2–4	30
< 2	7

### Effectiveness

Table 4 summarizes the outcome measures scores at baseline and end of study for the per-protocol population. Significant improvement was observed on both the ASRS and BRIEF-A questionnaires. Improvement in the ASRS was evident for the inattentive subscale, indicating reduced ADHD inattentive symptoms following intervention [*t* (94) = 0.12, *p* = 0.037]. Self-reports on the ASRS total score approached significance [*t* (94) = 1.87, *p* = 0.064], showing a 4% reduction in ADHD symptoms post-intervention. No significant effect was found on the ASRS hyperactive subscale [*t* (94) = 1.08, *ns*]. Improvement in the BRIEF-A was evident for the Metacognitive Index [*t* (92) = 2.22, *p* = 0.029]. There were no significant effects for the BRIEF-A Behavior Regulation index or the Global Executive Composite [*t* (92) = 0.73, *ns.,* and *t* (92) = 1.73, *ns.*, respectively].

**Table 4:** Outcome measures scores at baseline and end-of-study for the per-protocol population (*n* = 97).

	Baseline		End of study		*p*-value	Cohen’s d
	Mean	SE	Mean	SE		
ASRS – Inattentive	21.20	0.69	20.22	0.72	0.037	0.22
ASRS – Hyperactive	18.56	0.68	18.05	0.70	0.283	–
ASRS – Total Score	39.76	1.24	38.27	1.31	0.064	–
BRIEF – A – Behavioral Regulation Index	54.75	1.22	54.26	1.28	0.465	–
BRIEF – A – Metacognition Index	79.85	1.75	77.76	1.75	0.029	0.23
BRIEF – A – Global Executive Composite	134.60	2.69	132.02	2.75	0.088	–
CPT-3 – *d’*	50.03	0.90	47.95	1.06	0.027	0.23
CPT-3 – Omissions	48.39	0.88	47.86	0.82	0.562	–
CPT-3 – Commissions	52.26	0.92	49.81	1.15	0.004	0.30
CPT-3 – Hit Reaction Time	51.61	0.64	51.63	0.76	0.973	–
CPT-3 – Hit Reaction Time SD	45.13	0.86	45.68	0.88	0.522	–

ASRS, Adult ADHD Self-Report Scale; BRIEF-A, Behavior Rating Inventory of Executive Function-Adult Version; CPT-3, Conners’ Continuous Performance Test-3.

The effect of a two-month intervention on attentional control was tested using the CPT-3. Our findings indicate significant improvement in target detectability (i.e., d’; *t* (92) = 2.25, *p* = 0.027) and reduced incorrect responses to non-targets (i.e., commission errors; *t* (92) = 2.92, *p* = 0.004). All other CPT-3 measures were less sensitive to intervention-based changes in performance (Table 4).

Response to the intervention was assessed using the CGI-I. Participants were considered responders if they had a CGI-I score of “1” or “2” (very much improved or much improved, respectively). Sixty-two percent of the participants met the response criteria, having much or very much symptomatic improvement after using the Neuro-glasses.

No significant covariate effect was observed for demographic or baseline parameters such as gender, age, eyeglasses, learning disabilities, treatment naïve, and ASRS total score.

## Discussion

This study provides preliminary support for the safety and efficacy of a peripheral visual stimulation-based device in managing ADHD in adults. Reduction in ADHD symptoms was demonstrated by the ASRS inattentive subscale, reflecting the rated adult’s difficulty in maintaining attention for long periods, organizing/planning, paying attention to details, and committing reckless mistakes (31). No significant improvement was found on the ASRS hyperactive subscale. The reduced inattentive symptoms occurrence was consistent with an improvement in the metacognition index of the BRIEF-A questionnaire, evaluating the ability to self-manage tasks and monitor performance in different contexts (32). An objective, task-oriented assessment of attention and impulsivity, the CPT-3, revealed reduced difficulty in target detectability and commission errors, suggesting enhanced inhibition of response and attentional control. No significant reduction was found in the number of omission errors. This lack of findings is not unexpected given the low difficulty level of the task and the below-average omission baseline-score that falls outside the clinical range.

Despite the small effect sizes, the results are encouraging, given that the level of ADHD symptom manifestation in the study population was relatively mild, as reflected by the average ASRS baseline score. The clinical meaningfulness of the findings is further supported by the 62% of participants who positively responded to the intervention, as assessed by the CGI-I.

Assessment of the intervention adherence rate revealed that most participants (63%) wore the Neuro-glasses for at least 4 hours daily, whereas only 7% reported wearing the Neuro-glasses occasionally for up to 2 hours daily. These findings, together with the relatively low dropout rate (<10%), are of high importance, as they indicate that the intervention was well tolerated and accepted by ADHD adults. Moreover, the high adherence rate suggests that the effect of the Neuro-glasses is notable and meaningful for the user.

The current pilot study confirms that the Neuro-glasses hold minimal risks, as no clinically significant adverse events were associated with the intervention. Reports involved minor events of headache, dizziness, and eyestrain that were all transient, relatively mild and had not resulted in any discontinuations of the intervention.

The etiology of ADHD is related to various genetic and environmental factors (36). Though ADHD is not preventable or cured, the symptoms can be managed. Central nervous system stimulants are currently the best-known and most widely used ADHD treatment (6). Nevertheless, stimulants have a low adherence rate. The decision to discontinue treatment is primarily associated with medication-related side effects and ambivalence regarding chronic medication usage, especially at young ages (7, 8). In this study, demographic information revealed that 90% of the current study population reported a history of ADHD stimulant usage yet sought alternative treatment options. Alternative non-pharmacological interventions for ADHD include different variations of behavioral and cognitive treatments, Neurofeedback, sports, and diets/supplements. Though most of these treatments hold a more conservative, somewhat natural approach, they either fail to show solid empirical support for their efficacy or involve long-term treatment as they demand the acquisition of new coping skills. As such, some of those treatments are more commonly used as supplementation. Given the limitations of the medicated and non-medicated interventions, there is a pressing need for developing new treatment approaches for ADHD, such as the Neuro-glasses.

The Neuro-glasses presumably attenuate ADHD symptoms by stimulating the visual periphery. However, it is still unclear what is the mechanism underlying their effect. A possible explanation is that the Neuro-glasses might operate via the arousal system. Arousal is crucial for survival, as it regulates consciousness, attention, alertness, motivation, and information processing. Arousal regulation reflects the competence of the brain to deal with ongoing situational demands. Its dysregulation has been linked to psychiatric and physiological disorders and cognitive impairments (37–40). Relevant to this study, arousal dysregulation has been associated with ADHD pathology, reflecting naturally reduced arousal levels in ADHD patients (26, 37, 41). The hypo-arousal state, characterizing ADHD, was suggested to explain the deficit in sustained attention and vigilance as well as the hyperactive and impulsive autoregulatory behaviors, aiming to raise the arousal level (38–40, 42). Although somewhat speculative, we suggest here that the Neuro-glasses may act as a source of arousal. Previous studies have shown that peripheral stimuli external to a task often influence target identification and discrimination (20, 21, 43), decision-making (18), and visual search (19). This facilitative effect was reported to be more pronounced in adults with ADHD-like traits (22). The beneficial effect of extra-task stimulation on cognitive performance of ADHD patients is supported by several models of ADHD, including the optimal stimulation model (27), the cognitive-energetic model (26), and the moderate brain arousal model (MBA) (24). Interestingly, all models postulate that under certain circumstances, rather than being distracted by non-task stimulus, ADHDs benefit from its presence. They link this effect to the regulation of arousal levels and the extent to which variations in these factors can affect performance. It has been proposed that the external task stimuli facilitate performance by enhancing arousal to an optimal level. The resulting elevated arousal levels are assumed to increase the total attention capacity and narrow the focus of attention to the most dominant aspect of a given task, improving performance (26, 37, 38, 40, 42, 44).

While the Neuro-glasses are not an optical device by definition, they may operate via visual correction. A large body of evidence associates ADHD and problems of vision (9–11). ADHD patients were found to be comparable to controls with regard to visual acuity. However, they were more likely to have functional vision problems involving the alignment, focus, and movement of the eyes (10). As this may stress the importance of visual examinations in ADHD, it also implies that, in some cases, visual deficits may confound the diagnosis of ADHD. It is not unlikely to suggest that the Neuro-glasses correct visual deficits that mimic ADHD symptoms. As the incoming sensory inputs are of higher quality, the cognitive performance characterizing ADHD is improved. Future studies should involve a comprehensive visual examination to test the impact of the Neuro-glasses on visual problems.

While the results of this study are encouraging, we acknowledge certain important limitations. First, this is an uncontrolled study. Thus, we cannot rule out the contribution of placebo effects. Second, given the exploratory nature of the study, no primary endpoint was predetermined. Third, the current study population does not represent the high prevalence of comorbidities in the ADHD population and their effect on attention symptoms since ADHD adults having any psychiatric or neurological comorbidity were excluded from the study. Fourth, this study does not control for the possible confounding effects of visual problems and emotional or motivational factors that may affect the conclusive assessment of improvement. Fifth, due to the study design, it is essential to recognize that with regards to the CPT specifically, we cannot rule out a possible acute impact of wearing the Neuro-glasses, as opposed to effects caused by their regular use.

To conclude, Neuro-glasses provide a non-pharmacologically safe approach to managing ADHD in adults. These preliminary encouraging findings merit further research including future controlled studies evaluating the efficacy of a peripheral visual stimulation-based intervention in adults and possibly children with ADHD. Moreover, the current study provides important insights into the utility of visual stimulation as an intervention for treating ADHD and potentially for additional psychiatric and neurological disorders involving dysregulation of arousal levels.

## Data availability statement

The original data collected in the study is included in the article. Further inquiries can be directed to the corresponding author.

## Ethics statement

The studies involving humans were approved by Max Stern Yezreel Valley College Institutional Review Board. The studies were conducted in accordance with the local legislation and institutional requirements. The participants provided their written informed consent to participate in this study.

## Author contributions

YR: Conceptualization, Methodology, Project administration, Resources, Supervision, Writing – original draft. CG: Conceptualization, Investigation, Writing – review & editing. GV: Conceptualization, Investigation, Writing – review & editing. CB-M: Data curation, Formal analysis, Methodology, Project administration, Writing – review & editing. DG: Conceptualization, Data curation, Methodology, Software, Writing – review & editing. NM-K: Supervision, Writing – original draft. ZE: Conceptualization, Investigation, Supervision, Writing – review & editing.
